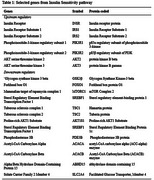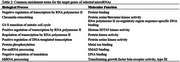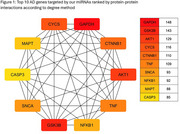# In Silico Identification of MicroRNAs Targeting Shared Pathways in Insulin Resistance and Alzheimer's Disease

**DOI:** 10.1002/alz70855_101481

**Published:** 2025-12-23

**Authors:** Tehniat Faraz Ahmed, Zeba Haque, Affan Ahmed, Jawwad us Salam, Shaheen Bhatty, Farina Hanif

**Affiliations:** ^1^ Dow University of Health Sciences, Karachi, Sindh, Pakistan

## Abstract

**Background:**

The unclear pathophysiology of Alzheimer's disease complicates treatment development. Risk factors like diabetes, metabolic syndrome, and APOE4 allele are closely linked to insulin resistance (IR), but mechanisms connecting these conditions are unclear. Epigenetic regulation through microRNAs has been implicated in the regulation of both IR and AD, yet no studies have identified microRNAs simultaneously targeting both pathways. This study aims to bioinformatically identify such microRNAs and analyze functional annotation of their target genes to uncover shared regulatory mechanisms linking IR and AD.

**Method:**

Using five prediction tools and two databases of experimentally validated microRNA‐gene interactions we identified microRNAs targeting twenty key IR genes in humans (Table‐1). We assigned a predicted score (P1) to each interaction, adding a point (E1) for experimental validation to calculate a cumulative score (C1) and its average (T1). We further analyzed microRNAs with T1 scores above 1.0 for interactions with AD pathway genes using KEGG analysis and constructed protein‐protein interaction network with STRING to identify hub proteins. We re‐evaluated the microRNAs for targeting these genes, calculating cumulative (C2) and total scores (T2). We performed functional annotation of target genes for microRNAs with the highest T2 scores using DAVID. Retrieving Gene Ontology terms for biological processes and molecular functions, we identified common terms to highlight shared regulatory mechanisms linking IR and AD.

**Result:**

AKT3 had the most microRNA targets among insulin sensitivity regulators, and miR‐15a‐5p scored the highest among the selected microRNAs with high T1‐scores. Altogether, these microRNAs targeted 299 AD‐related genes, with miR‐16‐5p targeting the most. The top 10 hub genes in the AD protein‐protein interaction network are shown in Figure 1. Rescoring based on AD targets revealed miR‐7‐5p, let‐7a‐5p, let‐7b‐5p, miR‐15b‐5p, miR‐16‐5p, miR‐17‐5p, and miR‐424‐5p as the highest‐ranking microRNAs based on total scores (T2). Target genes of these microRNAs were found enriched in negative regulation of transcription by RNA polymerase II, chromatin remodeling, and G1/S transition of the mitotic cell cycle (Table‐2). Molecular functions enriched included protein binding and protein serine/threonine kinase activity.

**Conclusion:**

We recommend studying the identified microRNAs as a pathogenic link between AD and IR and as early biomarkers of AD in IR patients.